# Retracted: Evaluation by an Aeronautic Dentist on the Adverse Effects of a Six-Week Period of Microgravity on the Oral Cavity

**DOI:** 10.1155/2021/9102451

**Published:** 2021-04-14

**Authors:** International Journal of Dentistry

*International Journal of Dentistry* has retracted the article titled “Evaluation by an Aeronautic Dentist on the Adverse Effects of a Six-Week Period of Microgravity on the Oral Cavity” [1] due to redundant publication with an article published earlier by two of the authors in 2009 [2] and other concerns with the ethics and reporting, as initially raised by Dr. Elisabeth Bik [3, 4]. Dr. Rai does not agree with retraction. Drs. Kaur and Foing did not respond.

## 1. Redundant Publication and Experimental Details

A 2009 article by the same group [2] should have been cited and discussed. The baseline results in Table 2 are identical, and the results in Table 1 are highly similar and some are identical: the baseline amylase means and the Cl and malonaldehyde means for during simulated microgravity vs. last day of HDT. The ranges/variances are the same at baseline for flow rate, Na, K, calcium, phosphate, protein, Cl, protein output, amylase, vitamin E, vitamin C, lactate dehydrogenase, MIP 1 alpha, malonaldehyde, 8-hydroxydeoxyguanosine, and thiocynate, and the ranges/variances are the same during simulated microgravity vs. last day of HDT for flow rate, Na, K, calcium, phosphate, protein, Cl, protein output, amylase, vitamin E, vitamin C, lactate dehydrogenase, MIP 1 alpha, malonaldehyde, 8-hydroxydeoxyguanosine, and thiocynate.

Dr. Rai said the studies had many of the same participants, i.e., volunteers who were members of the Joint Board of Research (JBR Institute of Health Education Research & Technology, India, the first affiliation of Dr. Rai), who were given the same diet and supplements. However, the age ranges do not overlap: 20 participants aged 18–22 in 2009 (though the abstract says 40 participants) versus 10 participants aged 22–30 in 2011. No information about a standard diet and supplements was included in the article or in the consent form and study protocol provided by Dr. Rai.

The exact protocol used in the study is unclear; the study protocol provided by Dr. Rai is brief and lacks additional detail compared to the article. The article implied that participants stayed in the head-down-tilt position for 8 hours, but Dr. Rai clarified that the participants were allowed to leave the position to go to the toilet. However, Dr. Bik noted that, in another study by the same group also published in 2011 [5], it was stated that participants stayed in the head-down-tilt position for 60 days, even during meals, showers, and toilet visits, and compliance was monitored by video. These protocols are therefore inconsistent.

There are further inconsistencies and missing information:

- Details of the protocol are missing from the 2011 article [1] compared to the 2009 article [2]. For example, the earlier article says the participants had eight hours head-down-tilt and then four hours recovery in a chair.
- The consent form says “about 20 people” would be recruited, whereas the study protocol says “ten to thirty healthy controls.”
- The protocol mentions “controls” rather than “participants.”
- The protocol discusses measuring heart rate variability during sleep, but sleep studies are not mentioned in the consent form or article.
- It is not stated when the study began and ended, what the roles of the authors were in the research, and whether anyone other than the authors contributed to the study.
- It is not clear whether the differences reported in Tables 1 and 2 were statistically significant nor whether the numbers in brackets in Table 1 are ranges or variances (e.g., 95% confidence intervals, standard error, or standard deviation).

Dr. Rai did not provide anonymised individual data points at all timepoints for all outcomes and measurements when this was requested.

## 2. Ethics

Dr. Rai provided a blank copy of the written informed consent document, which only briefly refers to what could be serious adverse effects (e.g., infection) without describing the likelihood or medical monitoring procedures. The consent form only refers to “the influence of isolated environment on human physiology and psychology,” not that participants would be subject to six weeks of head-down-tilt bed rest. The number is “IRB #2013,” which appears to correspond to a study done in 2013, not 2011.

The ethical approval document is signed by Prof. Suresh C. Anand, who has been a collaborator of Dr. Rai since 2006, when they were affiliated to the Government Dental College, and they were collaborators at the time of the study: this is not evidence of independent ethical approval. Because Prof. Anand was actively collaborating with Drs. Rai and Kaur and he was listed as the patron of the JBR Health Education and Research Organization, he should have recused himself from any ethical approval process at JBR.

The institutional review board (IRB) approval was given on January 5, 2010, but the study protocol cites two publications from 2010 (Rai et al. 2010 and Vetter et al. 2010); because the study protocol should have been approved by the IRB and then not altered, this is concerning. It is unclear which article is meant by Vetter et al., 2010, because there is no published fatigue scale by anyone by that name.

Dr. Rai said the participants were members of the Joint Board of Research, which was not disclosed in the article. Because Dr. Rai found and is president of this organisation, the use of these participants created a risk of coercion because they may have felt obliged to volunteer for this research. The 2006 Indian Council of Medical Research (ICMR) guidelines that applied to this research state “adequate justification is required for the involvement of participants such as prisoners, students, subordinates, employees, service personnel etc. who have reduced autonomy as research participants, since the consent provided may be under duress or various other compelling reasons” [6]. No such justification was provided. This is particularly relevant in the absence of independent ethics oversight.

The consent form says that no compensation would be paid in the event of injury. However, the ICMR guidelines [6] state “Research participants who suffer physical injury as a result of their participation are entitled to financial or other assistance to compensate them equitably for any temporary or permanent impairment or disability.”

## 3. Text Similarity

There is text overlap with four uncited articles [7–10], which should have been cited at their point of use. Their wording should not have been used without explicit quotation:

- 130 words from Heer, M., Boerger, A., Kamps, N. et al., “Nutrient supply during recent European missions,” Pflügers Arch - Eur J Physiol (2000) 441 (Suppl 1): R8. https://doi.org/10.1007/s004240000334
- 50 words from Stuempfle, K., and D. Drury. The Physiological Consequences of Bed Rest. Journal of Exercise Physiology online (June 2007) 10 (3): 32–41.
- 30 words from Olabi, A., Lawless, H., Hunter, J., Levitsky, D. and Halpern, B. (2002), The Effect of Microgravity and Space Flight on the Chemical Senses. Journal of Food Science, 67: 468–478. doi:10.1111/j.1365-2621.2002.tb10622.xhttps://onlinelibrary.wiley.com/doi/abs/10.1111/j.1365-2621.2002.tb10622.x
- 30 words from Elisabeth Blaber, Helder Marçal, and Brendan P. Burns, “Bioastronautics: The Influence of Microgravity on Astronaut Health,” Astrobiology 2010 10: 5, 463–473 https://www.liebertpub.com/doi/abs/10.1089/ast.2009.0415

## 4. Affiliations

The affiliations of Balwant Rai to the Kepler Space Institute and Jasdeep Kaur to KU Leuven (Catholic University, Leuven) are disputed by these institutions and should not have been used.
